# Anti-inflammatory Therapies for Coronary Heart Disease: A Systematic Review and Meta-Analysis

**DOI:** 10.3389/fcvm.2021.726341

**Published:** 2021-08-25

**Authors:** Haiming Wang, Min Jiang, Xin Li, Yunzhang Zhao, Junjie Shao, Zifan Liu, Lejian Lin, Qiang Xu, Lin Wang, Xuechun Lu, Haomin Zhang, Yundai Chen, Ran Zhang

**Affiliations:** ^1^Department of Cardiovascular Medicine, Chinese PLA General Hospital, Chinese PLA Medical School, Beijing, China; ^2^Department of Health Services, The First Medical Center of Chinese PLA General Hospital, Beijing, China; ^3^Department of Hematology, The Second Medical Center of Chinese PLA General Hospital, Chinese PLA Medical School, Beijing, China

**Keywords:** anti-inflammatory therapy, coronary heart disease, residual inflammation risk, major cardiovascular events, meta-analysis

## Abstract

**Background:** Anti-inflammatory therapy has been proposed as a promising treatment for coronary heart disease (CHD) that could reduce residual inflammation risk (RIR) and therefore major adverse cardiovascular events. We implemented a systematic review and meta-analysis of randomized controlled trials (RCTs) to assess the clinical benefits of anti-inflammatory agents in patients with CHD based on secondary cardiovascular prevention.

**Methods:** We systemically searched the PubMed, Embase, and Cochrane Library databases for RCTs (published between Jan 1, 1950, and June 1, 2021; no language restrictions) that focused on anti-inflammatory therapy for coronary heart disease. Our primary end points of interest were a composite of all-cause death, recurrent myocardial infarction and stroke. We processed pooled data using a random-effects model.

**Results:** Of 1497 selected studies, 18 studies with 67,449 participants met our inclusion criteria and were included in the present meta-analysis. Comparing anti-inflammatory agents with placebo, there was no significant decrease in risk of primary end points, secondary end points, all-cause mortality, cardiac mortality, recurrent myocardial infarction, stroke or revascularization. Further subgroup analysis indicated that anti-inflammatory agents led to a significant reduction in secondary end points (OR 0.87, CI 0.77–0.99; *P* = 0.03), recurrent myocardial infarction (OR 0.86, CI 0.78–0.95; *P* = 0.003) and revascularization (OR 0.81, CI 0.70–0.92; *P* = 0.001) in patients with stable CHD compared with placebo. Moreover, stable CHD patients had a lower propensity for recurrent myocardial infarction than acute coronary syndrome (ACS) patients when using anti-inflammatory agents (*P* = 0.03). The colchicine subgroup analysis showed that colchicine yielded a promising reduction in the primary end points (OR 0.81, CI 0.70–0.95; *P* = 0.009) compared with placebo. Anti-inflammatory agents were associated with a higher risk of infection (OR 1.13, CI 1.03–1.23; *P* = 0.007) and negligible effects on cancers (OR 0.98, CI 0.90–1.06; *P* = 0.61).

**Conclusion:** Anti-inflammatory agents appear to have beneficial effects in reducing the risk of recurrent myocardial infarction in patients with stable CHD, albeit at the cost of increased infection. Notably, colchicine demonstrates a promising cardioprotective effect with a lower incidence of major cardiovascular events and thus is a potential therapeutic strategy for stable CHD patients.

**Systematic Review Registration:** PROSPERO, identifier CRD42021245514.

## Introduction

Coronary heart disease (CHD) is a progressive clinical syndrome that is associated with an enhanced risk of fatal myocardial infarction, stroke, revascularization or cardiogenic death (1, 2). The available treatments recommended by the guidelines have not been reliably shown to further alter this clinical course (1, 3). Recent evidence has indicated that residual inflammatory risk (RIR) accelerates the progression of CHD by modulating immune cells and inflammatory cytokines (2, 4). A series of clinical studies have attempted to evaluate the efficacy of various anti-inflammatory agents in CHD patients (5, 6). The CANTOS trial (The Canakinumab Anti-inflammatory Thrombosis Outcomes Study) demonstrated the pivotal role of anti-inflammatory manipulation in improving cardiovascular outcomes by reducing the levels of interleukin-1β (IL-1β) and high-sensitivity C-reactive protein (hsCRP) (7, 8). The latest LoDoCo2 trial (Colchicine in Patients with Chronic Coronary Disease/Low-Dose Colchicine 2) suggested a lower risk of cardiovascular events with the administration of colchicine (9). We therefore sought to collect the latest clinical evidence and objectively assess the potential roles of anti-inflammatory therapy in CHD treatment. However, considering that previous studies of individual anti-inflammatory therapies failed to show distinctive improvements in major cardiovascular events (MACEs) (10), we performed a systematic review and meta-analysis of randomized controlled trials (RCTs) to investigate the effects of anti-inflammatory therapy on cardiovascular outcomes in patients with CHD.

## Materials and Methods

### Search Strategy and Selection Criteria

This systematic review and meta-analysis was performed in accord with the Preferred Reporting Items for Systematic Reviews and Meta-Analyses (PRISMA) statement (11). We systematically searched the Embase, PubMed and Cochrane Library databases for relevant studies published between Jan 1, 1950, and June 1, 2021, with no language restrictions. We used the following combined text and MeSH terms: “anti-inflammatory agents” and “myocardial ischemia.” We also performed a manual search of the references of relevant meta-analyses and systematic reviews for eligible studies.

### Study Selection and Data Extraction

RCTs comparing anti-inflammatory agents to placebo in CHD were considered eligible for inclusion. All pre-enrolled studies must contain at least one of the clinical outcomes, including all-cause mortality, cardiac mortality, recurrent myocardial infarction, revascularization and major adverse events (infections and cancers). Our primary end point of interest was a composite of all-cause death, recurrent myocardial infarction and stroke. The secondary end points included the components of the primary end points as well as revascularization. The exclusion criteria were as follows: observational and retrospective studies; RCTs that did not report the cardiovascular outcomes of interest; and RCTs that used nonsteroidal anti-inflammatory drugs or involved cardiopulmonary bypass therapy.

Two independent investigators (HW and MJ) screened the retrieved studies on the basis of titles and abstracts, and the remaining studies that satisfied the inclusion criteria were reviewed for full-text evaluation. Studies selected for subsequent detailed analysis and data extraction were analyzed by two investigators (HW and MJ). Disagreements were settled by a third investigator, if necessary (RZ). We drew the requisite data from each included study via a standardized data extraction form. Quality assessment of the studies was performed according to the PRISMA recommendations.

### Statistical Analysis

We assessed the clinical benefits of anti-inflammatory agents on nine categorical variable outcomes: primary end points, secondary end points, all-cause mortality, cardiac mortality, recurrent myocardial infarction, revascularization and major adverse events (infections and cancers). The pooled estimates were presented as odds ratios (ORs) with 95% confidence intervals (CIs) using a random-effects model on the basis of the DerSimonian–Laird method. Given the heterogeneity of the eligible studies and its underlying influences on beneficial effects, both the Cochran *Q-*test and *I*^2^-test were used to assess the magnitude of the heterogeneity between studies for each outcome. When the value of the *I*^2^-test was >50%, a study was considered to have high heterogeneity. Sensitivity analyses were performed to identify which studies had increased heterogeneity. In addition, a funnel plot of each study's effect size against the standard error was constructed to assess the potential publication bias, and the trim-and-fill test was used to estimate the effects of publication bias on the interpretation of the outcome. According to the type of CHD or the kind of anti-inflammatory agents, we implemented two subgroup analyses to discover potential clinical benefits. In this meta-analysis, we used Review Manager 5.3 and Stata 14.0 for statistical data processing.

## Results

### Results of the Literature Search and Study Characteristics

**Figure 1:**
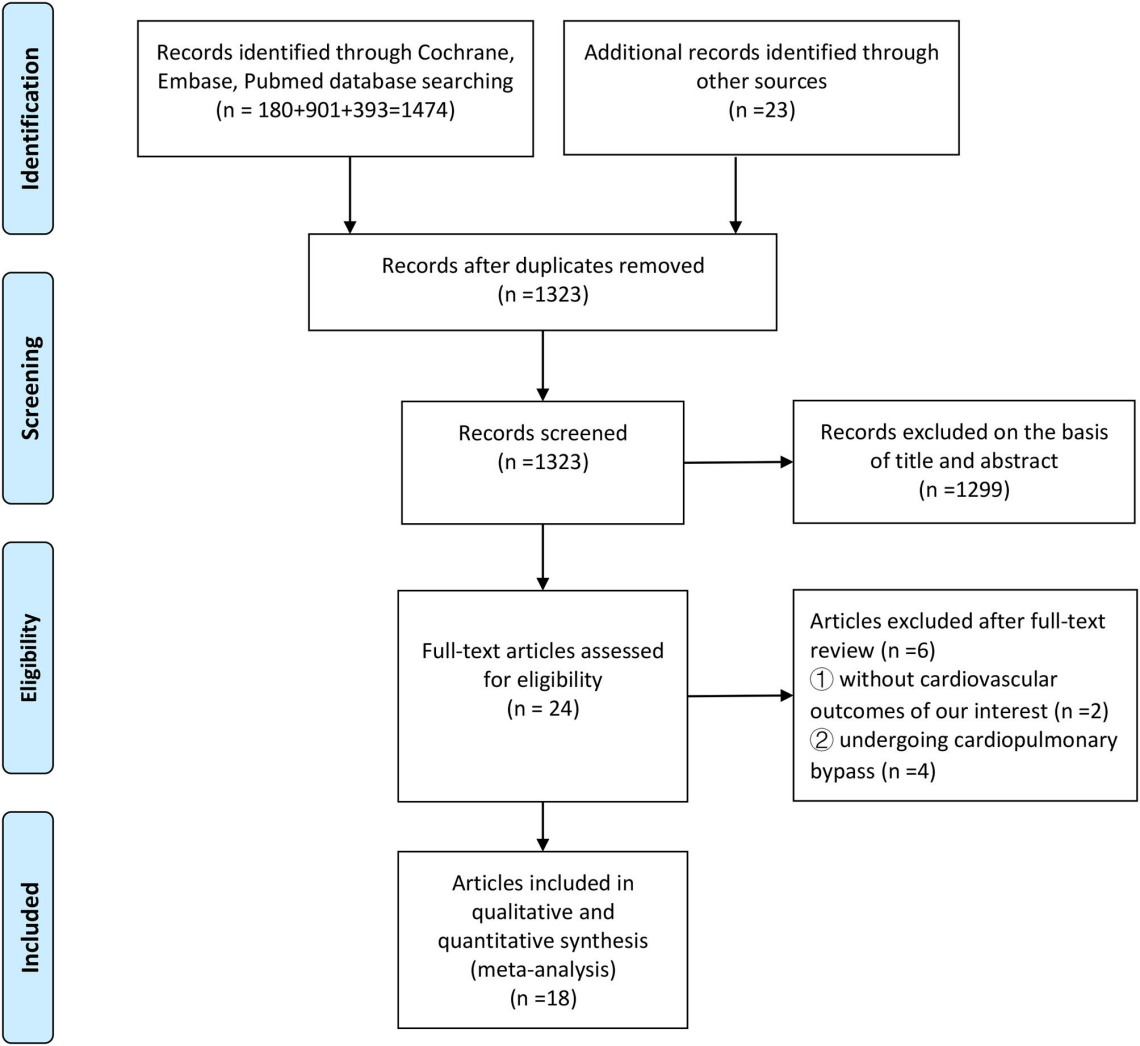
Study selection process.

The search strategy identified 1,497 studies, of which 18 studies with 67,449 participants met our inclusion criteria and were selected for the current meta-analysis after the three-level screening processes (Figure 1). The characteristics of all eligible studies that were published between 2003 and 2021 are summarized in Table 1. Nine anti-inflammatory agents, namely, colchicine, anakinra, darapladib, losmapimod, inclacumab, varespladib, pexelizumab, canakinumab, and methotrexate, were administered separately in the included studies. The weighted mean follow-up duration was 18.3 months (range 30 days−3.7 years), and the ages of the patients ranged from 51 to 74 years old. The majority of the patients were male, and all included patients had a high rate of typical risk factors, including hypertension, diabetes and hyperlipidemia. Of 18 studies reporting anti-inflammatory therapy, 5 studies—the LoDoCo trial, the STABILITY trial, the CANTOS trial, the CIRT trial and the LoDoCo2 trial—investigated patients with stable CHD, (7, 9, 12–14) and the remaining 13 trials recruited ACS patients (15–27). The assessment of risk of bias in this meta-analysis is shown in Supplementary Figure 1. All 18 RCTs reported adequate randomization, and only one yielded

**Table 1:** Characteristics of the included patients with CHD.

**Studies**	**Year**	**Type of disease**	**Anti-inflammatory agents**	**Patients of anti-inflammatory group**	**Patients of control group**	**Age of anti-inflammatory group (year)**	**Age of control group (year)**	**Median follow-up time**
**A**								
COMMA	2003	MI	Pexelizumab	281	271	58 (50–72)	61 (51–70)	6 months
APEX AMI	2007	STEMI	Pexelizumab	2,860	2,885	61 (51–71)	61 (52–71)	90 days
FRANCIS	2010	ACS	Varespladib	313	311	58.5 ± 10.3^#^	59.6 ± 10.5^#^	6 months
LoDoCo	2013	Stable CHD	Colchicine	282	250	66 ± 9.6^#^	67 ± 9.2^#^	3 years
SELECT-ACS	2013	NSTEMI	Inclacumab	176	175	59.8(53.4–68.7)	60.9 (54.4–66.6)	120 days
STABILITY	2014	Stable CHD	Darapladib	7,924	7,904	65.0 (59.0–71.0)	65.0 (59.0–71.0)	3.7 years
MRC-ILA	2014	NSTEMI	Anakinra	93	89	61.4 ± 11.7^#^	61.3 ± 12.3^#^	1 years
VISTA-16	2014	ACS	Varespladib	2,572	2,573	60.7 ± 9.8^#^	61.0 ± 10.0^#^	16 weeks
SOLID-TIMI 52	2015	ACS	Darapladib	6,504	6,522	64 (59–70)	64 (59–71)	2.5 years
VCU-ART and VCU-ART2	2015	STEMI	Anakinra	20	20	58 (51–65)	57 (48–60)	28 months
LATITUDE-TIMI 60	2016	MI	Losmapimod	1,731	1,758	66 (61–74)	67 (61–73)	12 weeks
CANTOS	2017	Stable MI	Canakinumab	2,263	3,344	61.1 ± 10.1^#^	61.1 ± 10.0^#^	48 months
VCUART3	2019	STEMI	Anakinra	31	35	55 (45–61)	56 (51–65)	12 months
CIRT	2019	Stable MI	Methotrexate	2,391	2,395	65.6 (59.7–71.8)	66.0 (59.8–71.7)	2.3 years
COLCOT	2019	MI	Colchicine	2,366	2,379	60.6 ± 10.7^#^	60.5 ± 10.6^#^	24 months
COPS	2020	ACS	Colchicine	396	399	59.7 ± 10.2^#^	60.0 ± 10.4^#^	12 months
COLCHICINE-PCI	2020	ACS	Colchicine	206	194	65.9 ± 9.9^#^	66.6 ± 10.2^#^	30 days
LoDoCo2	2020	Chronic CHD	Colchicine	2,762	2,760	65.8 ± 8.4^#^	65.9 ± 8.7^#^	28.6 months
**Studies**	**Male in anti-inflammatory group n (%)**	**Male in control group n (%)**	**Hypertension in anti-inflammatory group n (%)**	**Hypertension in control group n (%)**	**Diabetes in anti-inflammatory group n %)**	**Diabetes in control group n(%)**	**Hyperlipidemia in anti-inflammatory group n (%)**	**Hyperlipidemia in control group n (%)**
**B**								
COMMA	257 (91.5)	247 (91.1)	64 (22.7)	61 (22.5)	20 (7.1)	21 (7.7)	NA	NA
APEX AMI	2,169 (75.8)	2,251(78.0)	NA	NA	446 (15.6)	467 (16.2)	NA	NA
FRANCIS	230 (73.4)	236 (75.9)	271 (86.6)	274 (88.1)	84 (26.8)	87 (28.0)	120 (38.3)	109 (35.0)
LoDoCo	251 (89.0)	222 (88.8)	NA	NA	92 (32.6)	69 (27.6)	NA	NA
SELECT-ACS	89 (50.7)	90 (51.4)	NA	NA	26 (14.7)	24 (13.7)	NA	NA
STABILITY	6,463 (81.6)	6,398 (80.9)	NA	NA	2,664 (33.6)	2,687 (34.0)	2,646 (33.4)	2,786 (35.2)
MRC-ILA	63 (67.7)	67 (75.2)	31 (33.3)	29 (32.6)	NA	NA	27 (29.0)	28 (31.5)
VISTA-16	1,913 (74.4)	1,881 (73.1)	1,911 (75.2)	1,977 (77.8)	801 (31.3)	803 (31.3)	1,255 (49.3)	1,292 (50.9)
SOLID-TIMI 52	4,847 (74.5)	4,853 (74.4)	4,793 (73.7)	4,762 (73.0)	2,275 (35.0)	2,227 (34.1)	4,191 (64.5)	4,165 (63.9)
VCU-ART and VCU-ART2	18 (90.0)	12 (60.0)	14 (70.0)	12 (60.0)	4 (20.0)	5 (25.0)	13 (65.0)	14 (70.0)
LATITUDE-TIMI 60	1,231 (71.1)	1,226 (69.7)	1,268 (73.3)	1,276 (72.6%)	582 (33.6)	586 (33.3)	985 (56.9)	936 (53.2)
CANTOS	1,657 (73.2)	2,479 (74.1)	1,799 (79.5)	2,644 (79.1)	888 (39.2)	1,333 (39.9)	NA	NA
VCUART3	26 (83.9)	30 (85.7)	20 (64.5)	23 (65.7)	9 (29.0)	15 (42.9)	NA	NA
CIRT	1,930 (80.7)	1,958 (81.8)	2,153 (90.0)	2,169 (90.6)	788 (33.0)	823 (34.4)	NA	NA
COLCOT	1,894 (80.1)	1,942 (81.6)	1,185 (50.1)	1,236 (52.0)	462 (19.5)	497 (20.9)	NA	NA
COPS	322 (81.3)	310 (77.7)	201 (51.0)	199 (50.0)	75 (19.0)	76 (19.0)	180 (46.0)	185 (46.0)
COLCHICINE-PCI	193 (93.7)	181 (93.3)	192 (93.2)	175 (90.2)	114 (55.3)	117 (60.3)	182 (88.3)	173 (89.2)
LoDoCo2	2,305 (83.5)	2,722(98.6)	1,421 (51.4)	1,387 (50.3)	632 (22.9)	662 (24.0)	NA	NA

*COMMA, COMplement inhibition in Myocardial infarction treated with Angioplasty; APEX AMI, Assessment of Pexelizumab in Acute Myocardial Infarction trial; FRANCIS, Fewer Recurrent Acute Coronary Events With Near-Term Cardiovascular Inflammation*.

*Suppression; LoDoCo, Low-Dose Colchicine; SELECT-ACS, P-Selectin Antagonist Inclacumab on Myocardial Damage After Percutaneous Coronary Intervention for Non-ST-Segment Elevation Myocardial Infarction; STABILITY, Darapladib for Preventing Ischemic Events in Stable Coronary Heart Disease; MRC-ILA, Effect of Interleukin-1 Receptor Antagonist Therapy on Markers of Inflammation in Non-ST*.

*Elevation Acute Coronary Syndromes; VISTA-16, Vascular Inflammation Suppression to Treat Acute Coronary Syndromes for 16 Weeks; SOLID-TIMI 52, Stabilization of Plaque using Darapladib-Thrombolysis in Myocardial Infarction 52; VCU-ART, Virginia Commonwealth University-Anakinra Remodeling Trial; LATITUDE-TIMI 60, LosmApimod To InhibiT p38 MAP kinase as a therapeUtic target and moDify outcomes after an acute coronary syndrome; CANTOS, Canakinumab. Anti-Inflammatory Thrombosis Outcome Study; VCUART3, Virginia Commonwealth University-Anakinra Remodeling Trial-3; CIRT, Cardiovascular Inflammation Reduction Trial; COLCOT, Colchicine Cardiovascular Outcomes Trial; COPS, Colchicine in Patients with Acute Coronary Syndrome; COLCHICINE-PCI, Effects of Acute Colchicine Administration Prior to Percutaneous Coronary Intervention; LoDoCo2, Colchicine in Patients with Chronic Coronary Disease;*

# *denotes mean (SD); otherwise, they are number (percentage). Range values are median (interquartile range)*.

incomplete outcome data.

### Clinical Efficacy Endpoints

**Figure 2:**
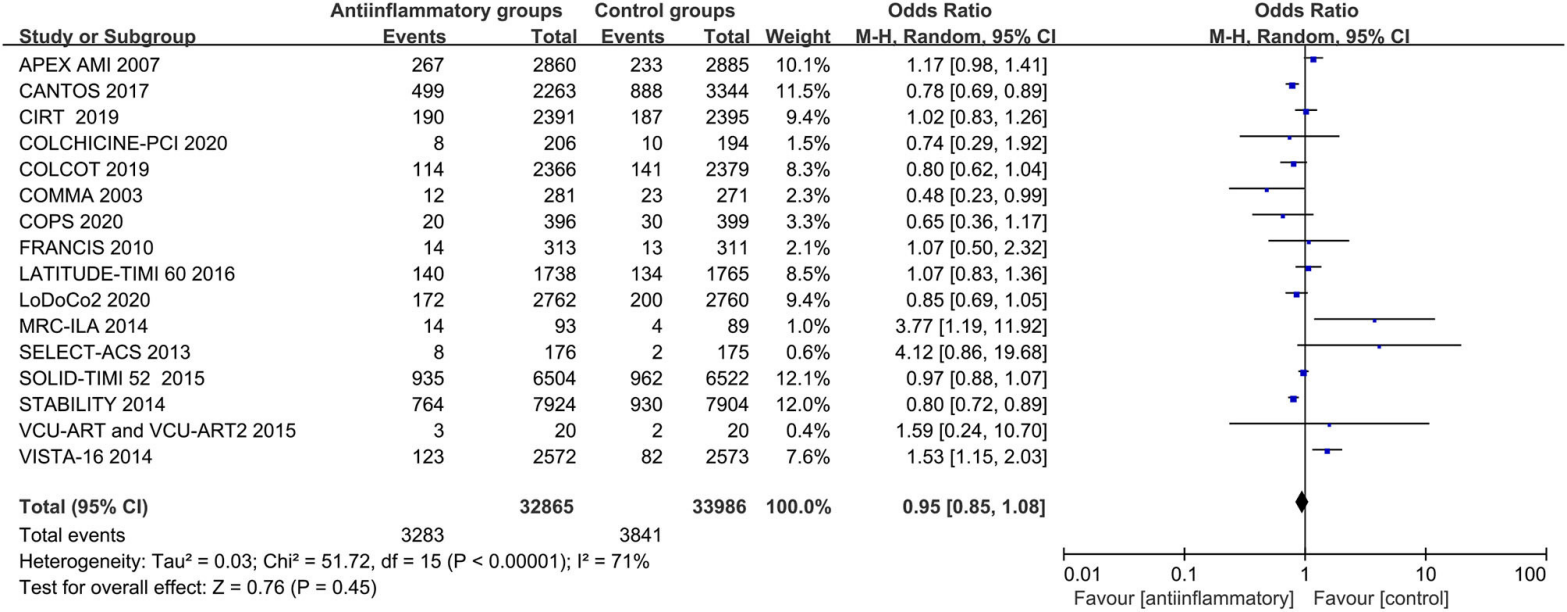
Forest plots of studies evaluating primary end points in patients receiving anti-inflammatory agents vs. placebo.

In a pooled analysis of all 18 studies, anti-inflammatory therapy did not statistically show a greater reduction in the primary end points (OR 0.99, CI 0.88–1.13; *P* = 0.92), secondary end points (OR 0.92, CI 0.82–1.04; *P* = 0. 02), all-cause mortality (OR 1.02, CI 0.93–1.11; *P* = 0.73), cardiac mortality (OR 0.94, CI 0.86–1.03; *P* = 0. 21), recurrent myocardial infarction (OR 0.99, CI 0.85–1.14; *P* = 0.86), stroke (OR 0.96, CI 0.84–1.10; *P* = 0.57) or revascularization (OR 0.87, CI 0.74–1.02; *P* = 0.09) than other therapeutic approaches, with different degrees of significant between-study heterogeneity (Figure 2 and Supplementary Figures 2–7). Visual inspection of the funnel plots intuitively demonstrated obvious graphical asymmetries, and the trim-and-fill test indicated that these publication biases did not impact the estimates (Supplementary Figure 8). Further sensitivity analysis showed that the results were similar when each study was individually excluded.

### Subgroup Analyses

#### Different Efficacies of Anti-inflammatory Agents Between Stable CHD and ACS

**Figure 3:**
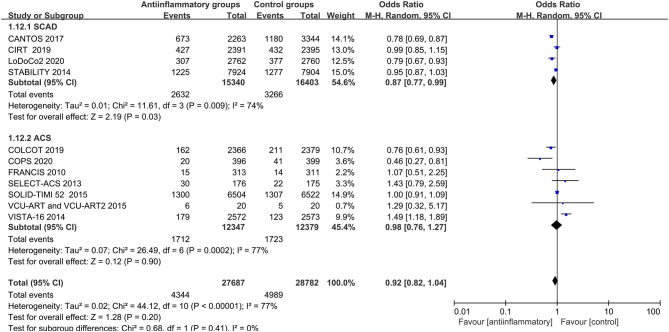
Forest plots of studies evaluating secondary end points in patients with stable CHD vs. ACS.

**Figure 4:**
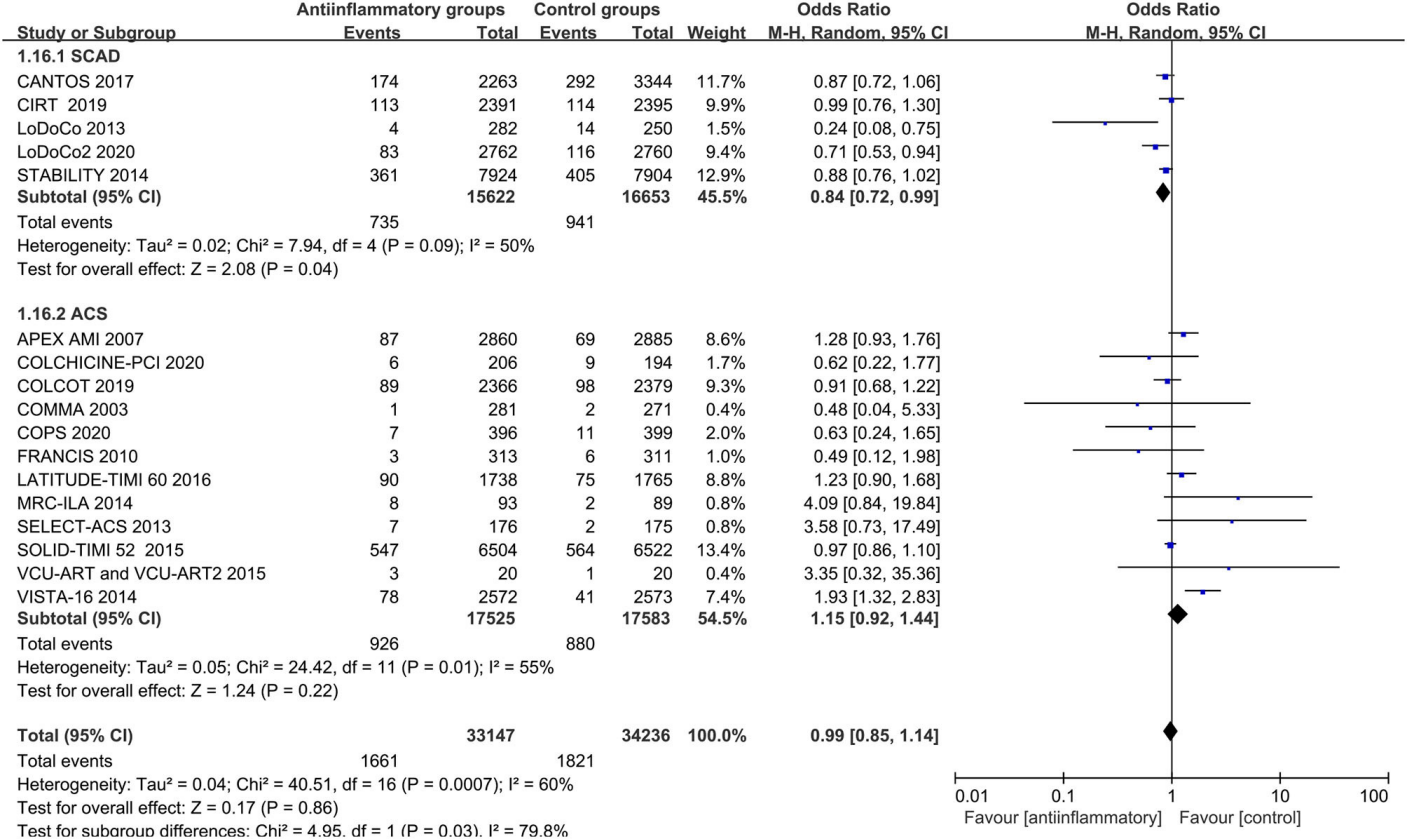
Forest plots of studies evaluating recurrent myocardial infarction in patients with stable CHD vs. ACS.

**Figure 5:**
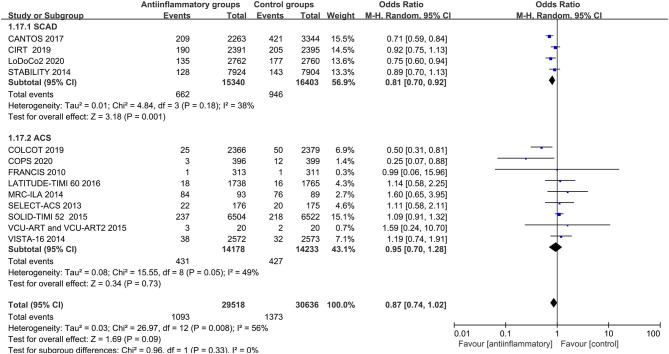
Forest plots of studies evaluating revascularization in patients with stable CHD vs. ACS.

Anti-inflammatory agents led to a significant reduction in secondary end points (OR 0.87, CI 0.77–0.99; *P* = 0.03), recurrent myocardial infarction (OR 0.86, CI 0.78–0.95; *P* = 0.003) and revascularization (OR 0.81, CI 0.70–0.92; *P* = 0.001) compared with placebo in patients with stable CHD, with different degrees of significant between-study heterogeneity (Figures 3–5). Visual inspection of their funnel plots showed that no publication biases were evident. Additionally, stable CHD patients had a lower propensity for recurrent myocardial infarction than the ACS patients when using anti-inflammatory drugs (*P* = 0.03; Table 2). No significant differences in primary end points, all-cause mortality, cardiac mortality or stroke were observed in this subgroup analysis (Supplementary Figures 9–12).

**Table 2:** Different efficacies of anti-inflammatory agents between stable CHD and ACS.

	**Stable CHD**	**ACS**	***P*-value**
Primary end points	0.93 [0.78, 1.10]	1.04 [0.87, 1.24]	0.38
Secondary end points	0.87 [0.77, 0.99]	0.98 [0.76, 1.27]	0.41
All-cause mortality	1.01 [0.93, 1.11]	1.02 [0.85, 1.22]	0.97
Cardiac mortality	0.96 [0.85, 1.08]	0.92 [0.79, 1.07]	0.65
Stroke	0.91 [0.77, 1.08]	1.01 [0.80, 1.27]	0.5
Recurrent myocardial infarction	0.84 [0.72, 0.99]	1.15 [0.92, 1.44]	0.03^*^
Revascularization	0.81 [0.70, 0.92]	0.95 [0.70, 1.28]	0.33

* *Denotes P < 0.05 for the comparison*.

#### Efficacy of Colchicine Use

**Figure 6:**
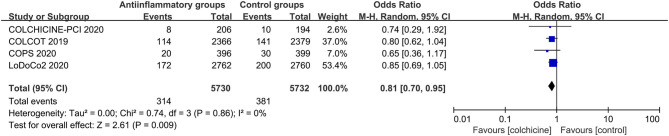
Forest plots of studies evaluating primary end points in patients receiving colchicine vs. placebo.

The subgroup analysis showed that colchicine yielded a promising reduction in the primary end points (OR 0.81, CI 0.70–0.95; *P* = 0.009) compared with placebo, with no significant between-study heterogeneity (Figure 6 and Supplementary Figure 13).

### Major Adverse Events

**Figure 7:**
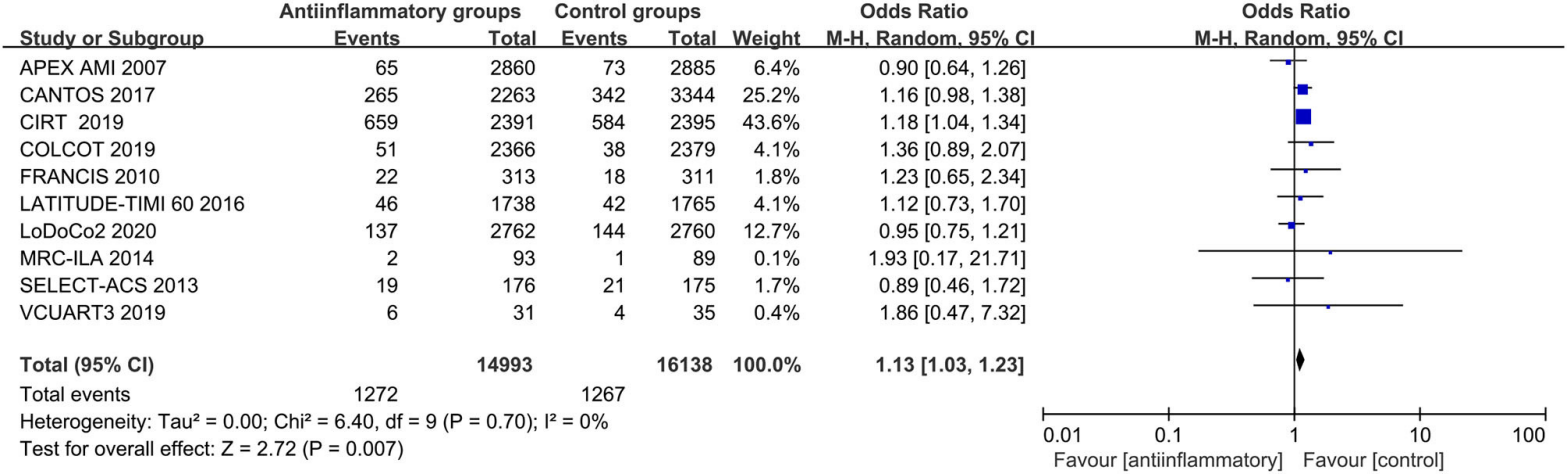
Forest plots of studies evaluating infections in patients receiving anti-inflammatory agents vs. placebo.

**Figure 8:**
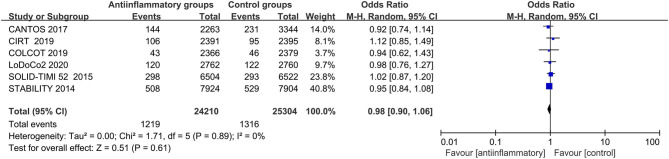
Forest plots of studies evaluating cancers in patients receiving anti-inflammatory agents vs. placebo.

Anti-inflammatory agents were associated with a higher risk of infections (OR 1.13, CI 1.03–1.23; *P* = 0.007) and had negligible effects on cancers (OR 0.98, CI 0.90–1.06; *P* = 0.61), with no significant between-study heterogeneity (Figures 7, 8 and Supplementary Figures 14, 15).

## Discussion

In this systematic review and meta-analysis, we showed that anti-inflammatory agents yield mild cardiovascular protective effects but result in an increased risk of infections. However, further subgroup analysis demonstrated that multiple anti-inflammatory agents reduce the risk of recurrent myocardial infarction and revascularization in stable CHD. Considering the different subtypes of the disease, there was a significant reduction in recurrent myocardial infarction in stable CHD patients compared with the ACS patients. Additionally, among nine anti-inflammatory agents, colchicine had pronounced cardiovascular benefits among patients with all kinds of CHD. Therefore, these data suggest that selective anti-inflammatory therapy is promising in the management of CHD.

### Current Evidence of Anti-inflammatory Therapy for CHD

The attainment of cardiovascular benefits is compromised by the limitations of available treatment for CHD (1, 2, 8, 28). Although low-density lipoprotein-cholesterol lowering and antiplatelet therapies have been widely recommended by guidelines and administered in clinical practice, more than half of CHD patients have a high level of hsCRP, a direct metric of systemic inflammation, to drive the natural history of this disease (2, 29, 30). Persistent residual inflammatory risk destabilizes atherosclerotic plaques and exacerbates the risk of MACEs (2). In the FOURIER (Further Cardiovascular Outcomes Research With PCSK9 Inhibition in Subjects With Elevated Risk) trial, patients with CHD were more prone to develop adverse cardiovascular events when proprotein convertase subtilisin-kexin type 9 (PCSK9) inhibitors were used to reduce low-density lipoprotein cholesterol (LDL-C) to a lower level but hsCRP levels exceeded the upper limit of normal (28, 31). In this context, anti-inflammatory therapy emerges as a novel strategy that can make further progress against this residual burden while alleviating long-term cardiovascular risk (29). As anticipated, both the CANTOS trial and the LoDoCo2 trial confirmed the beneficial effects of anti-inflammatory therapy with canakinumab and colchicine (7, 9). However, controversies regarding anti-inflammatory therapy still exist (10). In this meta-analysis, we did not find benefits of anti-inflammatory therapy in all CHD patients. Indeed, it is possible that the pooled OR may be skewed by a few potential chance findings in the present meta-analysis. Importantly, this inconsistency is mainly because the 18 eligible studies differ in several ways, including sample size differences and evaluation of clinical outcomes in individual studies, the time course of anti-inflammatory agents, and background oral secondary prevention medications (5, 10, 32). The between-study heterogeneity that adequately reflected these differences in study design were not attenuated. Future high-quality studies with larger samples should be conducted to obtain accurate estimates of the efficacy of anti-inflammatory agents. Collectively, more evidence is needed before anti-inflammatory therapy changes current clinical practice (10).

To minimize this heterogeneity in the included studies, we conducted additional subgroup analyses by modifying the inclusion criteria and excluding studies with different outcome definitions. Of particular interest, the five studies recruiting patients with stable CHD showed that substantial cardiovascular benefits were primarily attributed to risk reductions of 14, 19, and 13% for recurrent myocardial infarction, revascularization and secondary end points, respectively. This finding was consistent with a recently published meta-analysis focusing on stable CHD patients (5). These data could be interpreted by the pathogenesis of coronary atherosclerotic plaques that consists of a large lipid core and thin fibrous cap involving activated immune cells and inflammatory cytokines (2, 33–35). In the phase of plaque progression, anti-inflammatory therapy targeting inflammatory factors renders patients more susceptible to stabilization (2, 35, 36). When ACS occurs, immune cells and inflammatory cytokines are released from the plaque into the blood or depleted (36), which results in the loss of targets for anti-inflammatory agents. Furthermore, in stable CHD, the reserved microvascular system, intact myocardium and intervenable inflammatory pathways make anti-inflammatory therapy much more feasible and efficient than ACS (1, 5). Therefore, anti-inflammatory therapy is more likely to be beneficial in the early or stable stage of CHD.

The striking result was the CANTOS trial, in which canakinumab therapy successfully reduced cardiovascular risk, which indicates that the key inflammatory targets are most likely focused on the IL-1β to IL-6 to CRP pathway (2, 7, 8). In theory, colchicine, a widely available antitubulin agent, can irreversibly suppress the NLRP3 inflammasome and induce neutrophil dysfunction, and it therefore reduces circulatory levels of IL-1β, IL-6 and CRP (2, 6, 32, 37). Four eligible RCTs compared colchicine with placebo (9, 14, 15, 18, 26). Our data showed that colchicine reduces the risk of cardiovascular events by 19%, which was consistent with a recently published study of colchicine for secondary prevention of cardiovascular diseases (1). However, none of these 4 RCTs used hsCRP as an indicator for therapeutic effect evaluation or CT scans to assess plaque progression. Whether the anti-inflammatory benefits of colchicine are independent of serum lipid lowering remains uncertain (38). A previous study recruiting primary biliary cirrhosis patients showed a decline in the level of oxidized LDL-C after the administration of colchicine (38, 39). The mechanism of the cardiovascular benefit from colchicine needs further evaluation. Most other anti-inflammatory agents showed no significant cardiovascular benefits. This may be caused by different mechanisms and administration approaches of anti-inflammatory agents (10), and anti-inflammatory therapy focuses on regulating the immune balance rather than completely suppressing the inflammatory response (2, 5).

### Safety of Anti-inflammatory Therapy

There is a strong association between anti-inflammatory agents and the impaired host defense system (2, 7). The pooled analyses of all included studies showed that anti-inflammatory agents result in a 14% increase in the risk of infections. Secondary pneumonia was the most common; therefore, early monitoring and preventive antibiotic therapy could reconcile this treatment dilemma (2, 5). In addition, our data showed that anti-inflammatory agents conferred no significant risk of new incident cancers. This may indicate that drug-induced immunosuppression is not sufficient to promote tumorigenesis. In contrast, the CANTOS trial demonstrated a significant reduction in lung cancers by canakinumab (7). Ongoing studies are exploring the potential effects of canakinumab on lung cancer patients (29). For colchicine with the most clinical evidence of anti-inflammatory properties, it mainly increases the incidence of diarrhea, which is reversible with drug discontinuation (40). Moreover, combined administration of colchicine and statins in the LoDoCo-2 and COLCOT trials also exhibit no apparent increase in myopathy or rhabdomyolysis (40).

### Limitations

There were several limitations in this study. The mean follow-up duration of all RCTs was 18.3 months, and the minimum period was 30 days. The long-term outcome of anti-inflammatory therapy needs further evidence. Second, raw patient-level data of all selected studies could not be obtained; hence, only modified study-level data were extracted and reanalyzed in the present meta-analysis. Although the assessable end points of interest in this meta-analysis were objective in the recruited studies, some studies were still accompanied by a risk of bias, such as incomplete research data. Meanwhile, we should keep in mind that the implementation of revascularization in CHD patients in all eligible studies depends on the various indications practiced at different hospitals, which renders the decreased risk of revascularization as the softest of all cardiovascular events. Third, the ideal time course and doses of agents have not yet been confirmed in recent clinical trials (10). Whether the timing of drug administration is as early as possible remains uncertain. Finally, 7 trials recruited fewer than 300 people; thus, type I error and small sample size bias should be fully considered (14, 18, 19, 21, 23, 24, 27). More large-scale clinical trials are needed to further verify the effects of anti-inflammatory agents in CHD patients.

## Conclusion

Anti-inflammatory therapy reduces the risk of recurrent myocardial infarction in patients with stable CHD. Although uncertainties still exist, the addition of anti-inflammatory therapy to standard medical therapy in patients with CHD is promising for improving long-term cardiovascular outcomes.

## Data Availability Statement

The original contributions presented in the study are included in the article/Supplementary Material, further inquiries can be directed to the corresponding author/s.

## Author Contributions

HW, MJ, YC, and RZ contributed to the study conception and design, and writing the manuscript. HW, MJ, XLi, YZ, and ZL performed data collection and analysis. HW, MJ, RZ, LL, QX, LW, XLu and HZ commented on the research design, data analysis, writing the manuscript, and supervision of the study. All authors contributed to the article and approved the submitted version.

## Conflict of Interest

The authors declare that the research was conducted in the absence of any commercial or financial relationships that could be construed as a potential conflict of interest.

## Publisher's Note

All claims expressed in this article are solely those of the authors and do not necessarily represent those of their affiliated organizations, or those of the publisher, the editors and the reviewers. Any product that may be evaluated in this article, or claim that may be made by its manufacturer, is not guaranteed or endorsed by the publisher.

## Supplementary Material

Supplementary Figure 1 Bias risk assessment of the studies.

Supplementary Figure 2 Forest plots of studies evaluating secondary end points in patients receiving anti-inflammatory agents vs. placebo.

Supplementary Figure 3 Forest plots of studies evaluating all-cause mortality in patients receiving anti-inflammatory agents vs. placebo.

Supplementary Figure 4 Forest plots of studies evaluating cardiac mortality in patients receiving anti-inflammatory agents vs. placebo.

Supplementary Figure 5 Forest plots of studies evaluating recurrent myocardial infarction in patients receiving anti-inflammatory agents vs. placebo.

Supplementary Figure 6 Forest plots of studies evaluating stroke in patients receiving anti-inflammatory agents vs. placebo.

Supplementary Figure 7 Forest plots of studies evaluating revascularization in patients receiving anti-inflammatory agents vs. placebo.

Supplementary Figure 8 Funnel plots of odds ratios and standard errors to assess the publication bias of anti-inflammatory agents vs. placebo in primary end points (A), secondary end points (B), all-cause mortality (C), cardiac mortality (D), recurrent myocardial infarction (E), stroke (F) and revascularization (G).

Supplementary Figure 9 Forest plots of studies evaluating primary end points in patients with stable CHD vs. ACS.

Supplementary Figure 10 Forest plots of studies evaluating all-cause mortality in patients with stable CHD vs. ACS.

Supplementary Figure 11 Forest plots of studies evaluating cardiac mortality in patients with stable CHD vs. ACS.

Supplementary Figure 12 Forest plots of studies evaluating stroke in patients with stable CHD vs. ACS.

Supplementary Figure 13 Funnel plots of odds ratios and standard errors to assess the publication bias of colchicine vs. placebo in primary end points.

Supplementary Figure 14 Funnel plots of odds ratios and standard errors to assess the publication bias of anti-inflammatory agents vs. placebo in infections.

Supplementary Figure 15 Funnel plots of odds ratios and standard errors to assess the publication bias of anti-inflammatory agents vs. placebo in cancers.
