# Supplementary figures and images for: Anti-inflammatory Therapies for Coronary Heart Disease: A Systematic Review and Meta-Analysis
Source: Front Cardiovasc Med. 2021 Aug 25;8:726341. doi: 10.3389/fcvm.2021.726341 (PMC8424052; doi:10.3389/fcvm.2021.726341)

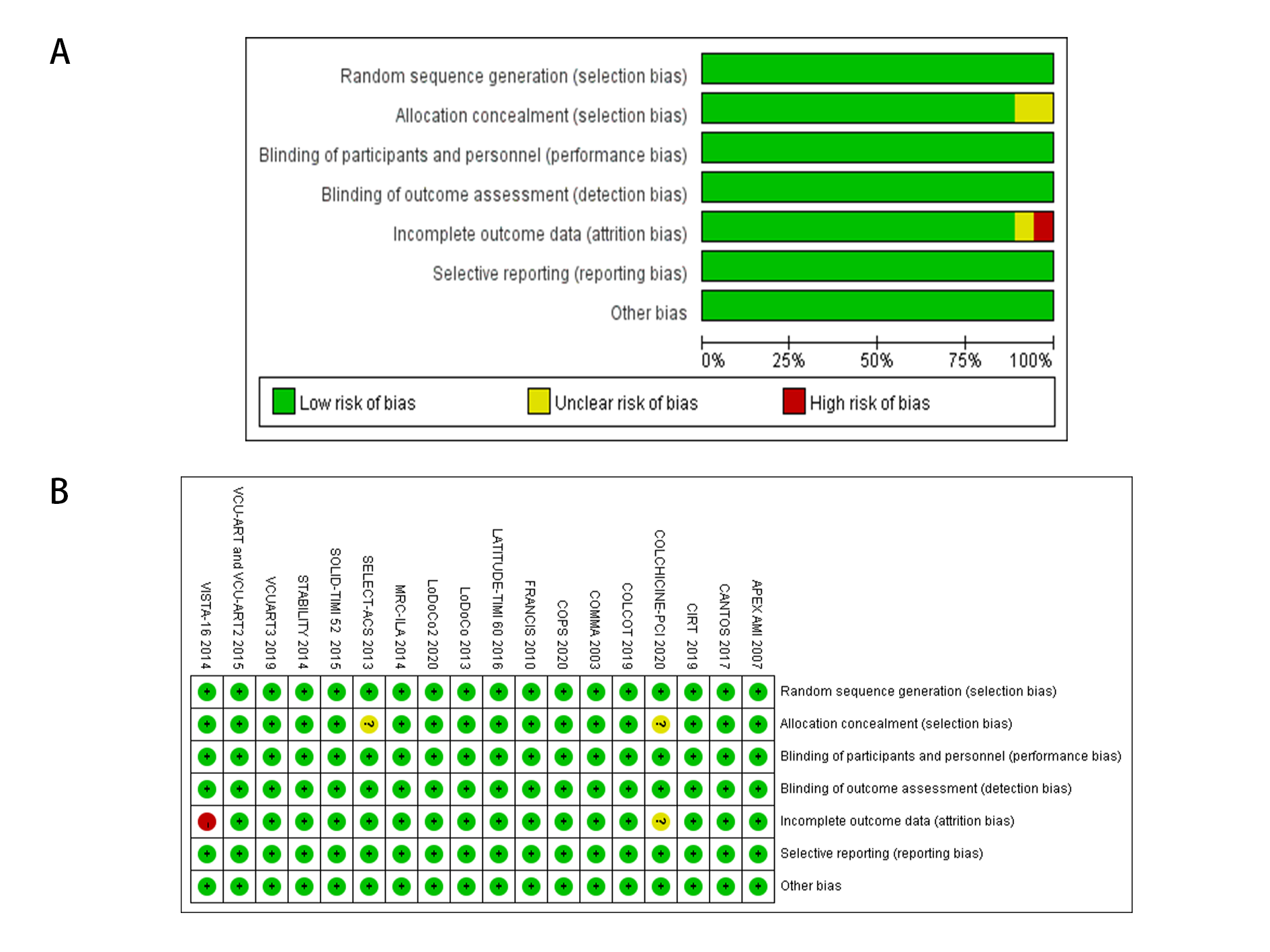

Supplement: Supplementary Figure 1 — Bias risk assessment of the studies. [file Image_1.TIF]

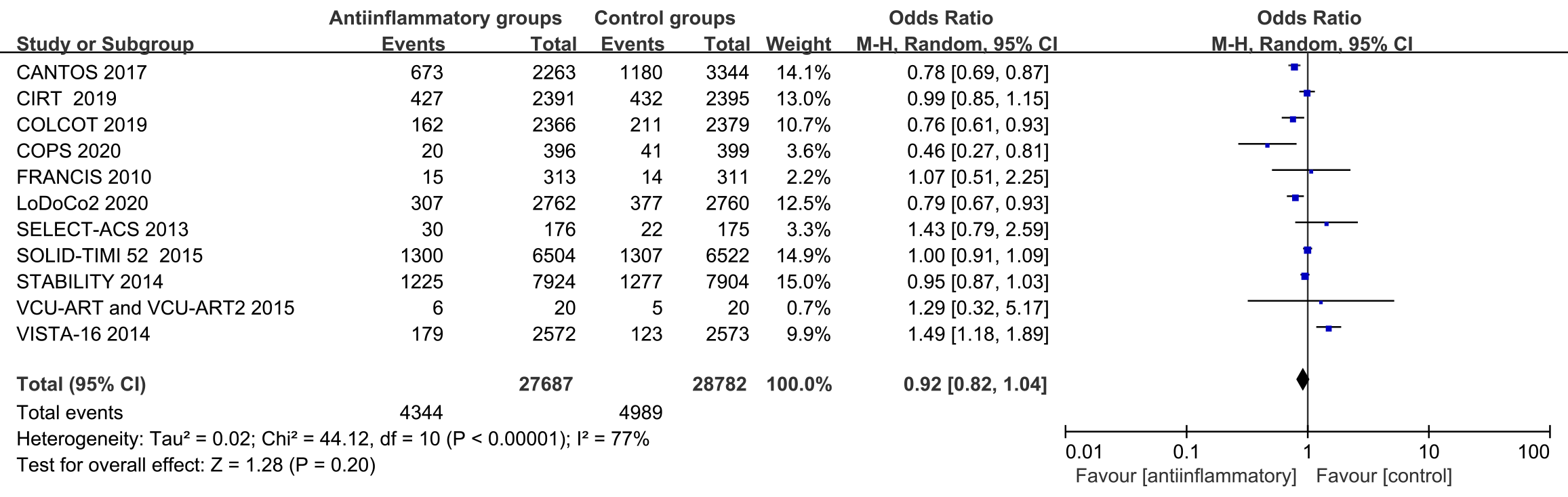

Supplement: Supplementary Figure 2 — Forest plots of studies evaluating secondary end points in patients receiving anti-inflammatory agents vs. placebo. [file Image_2.TIF]

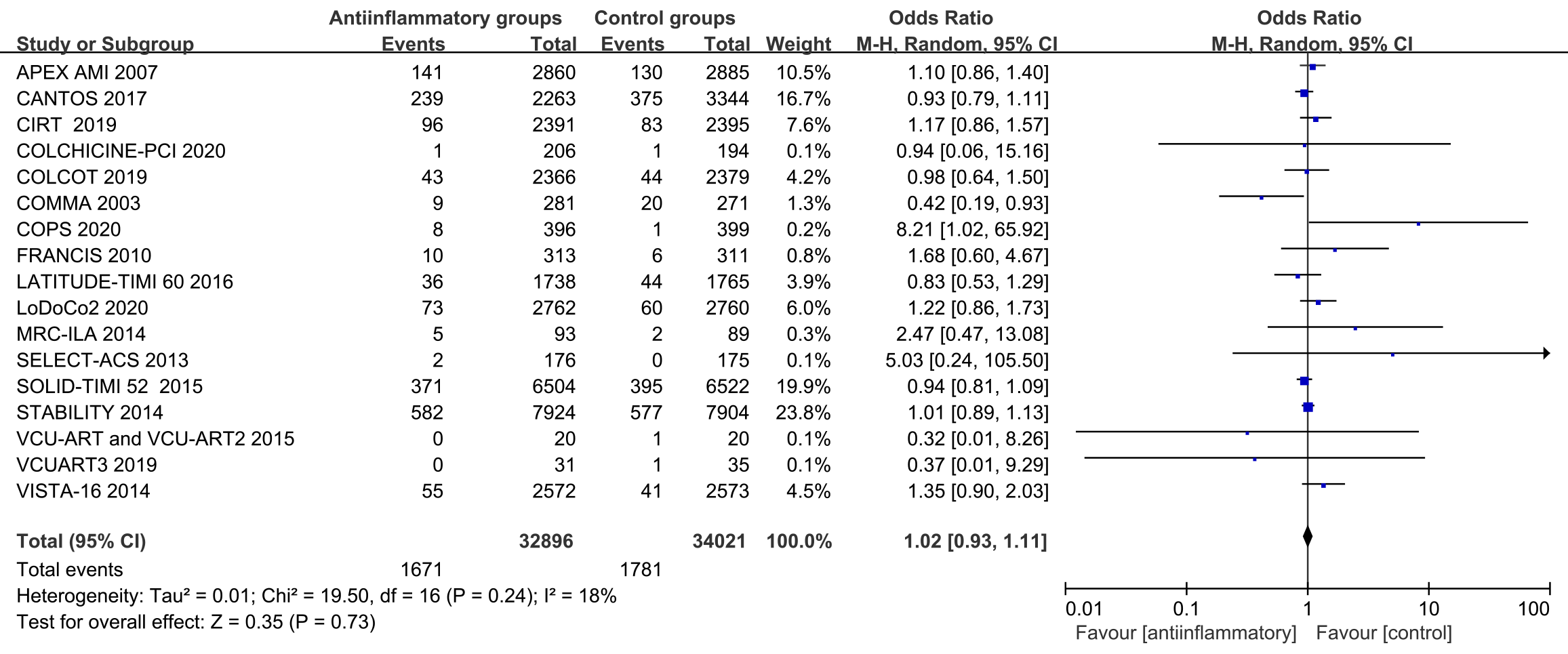

Supplement: Supplementary Figure 3 — Forest plots of studies evaluating all-cause mortality in patients receiving anti-inflammatory agents vs. placebo. [file Image_3.TIF]

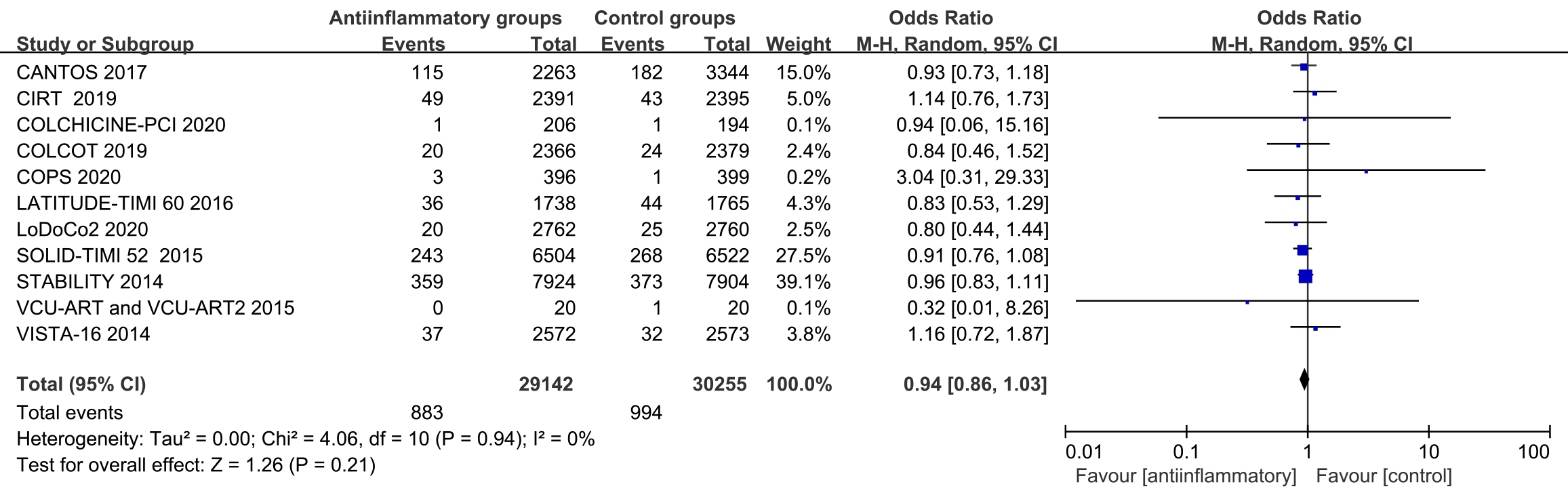

Supplement: Supplementary Figure 4 — Forest plots of studies evaluating cardiac mortality in patients receiving anti-inflammatory agents vs. placebo. [file Image_4.TIF]

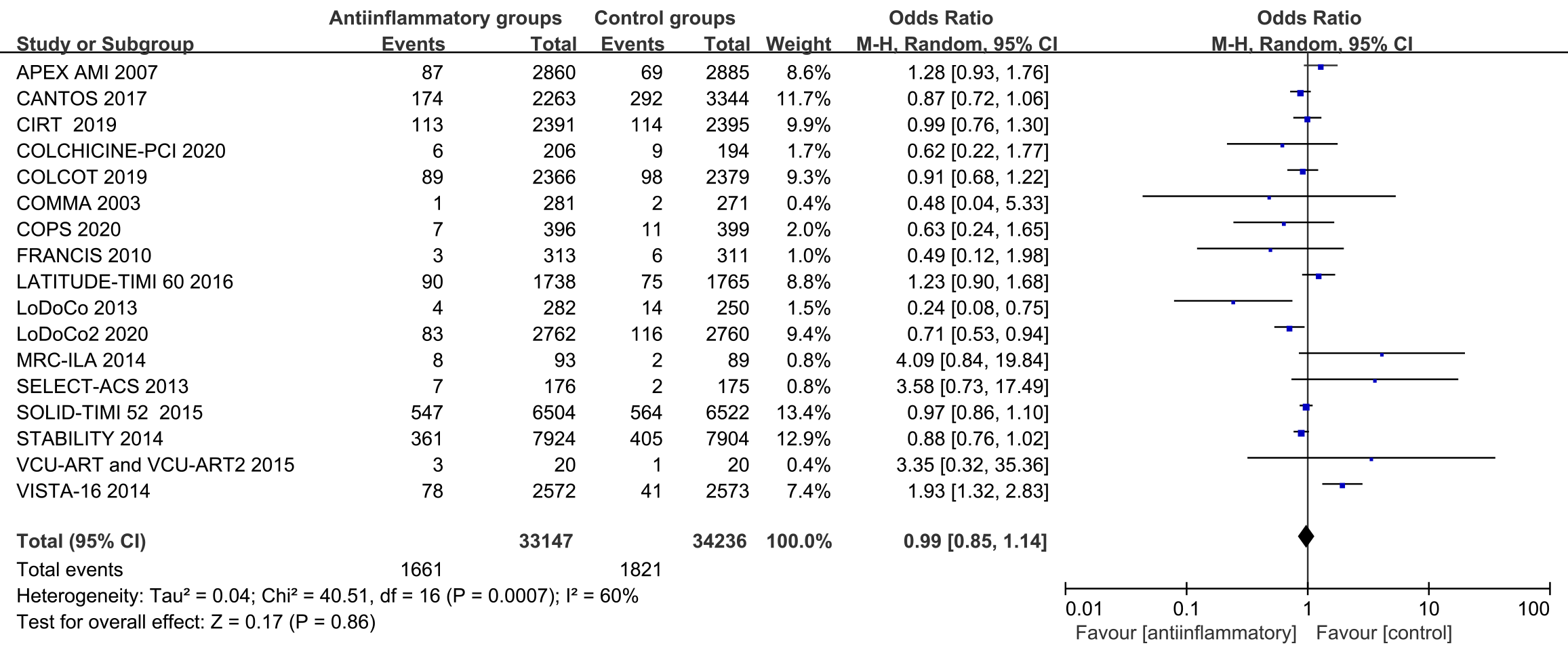

Supplement: Supplementary Figure 5 — Forest plots of studies evaluating recurrent myocardial infarction in patients receiving anti-inflammatory agents vs. placebo. [file Image_5.TIF]

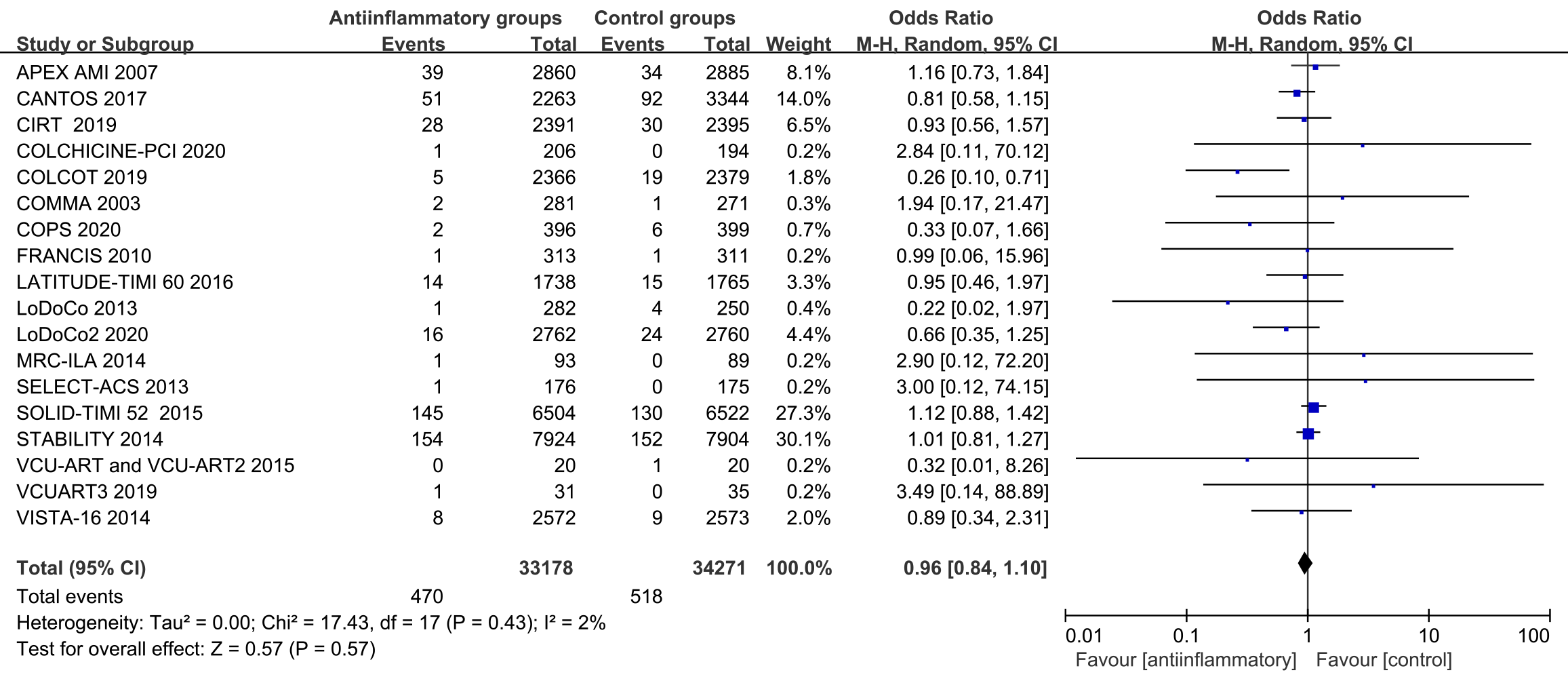

Supplement: Supplementary Figure 6 — Forest plots of studies evaluating stroke in patients receiving anti-inflammatory agents vs. placebo. [file Image_6.TIF]

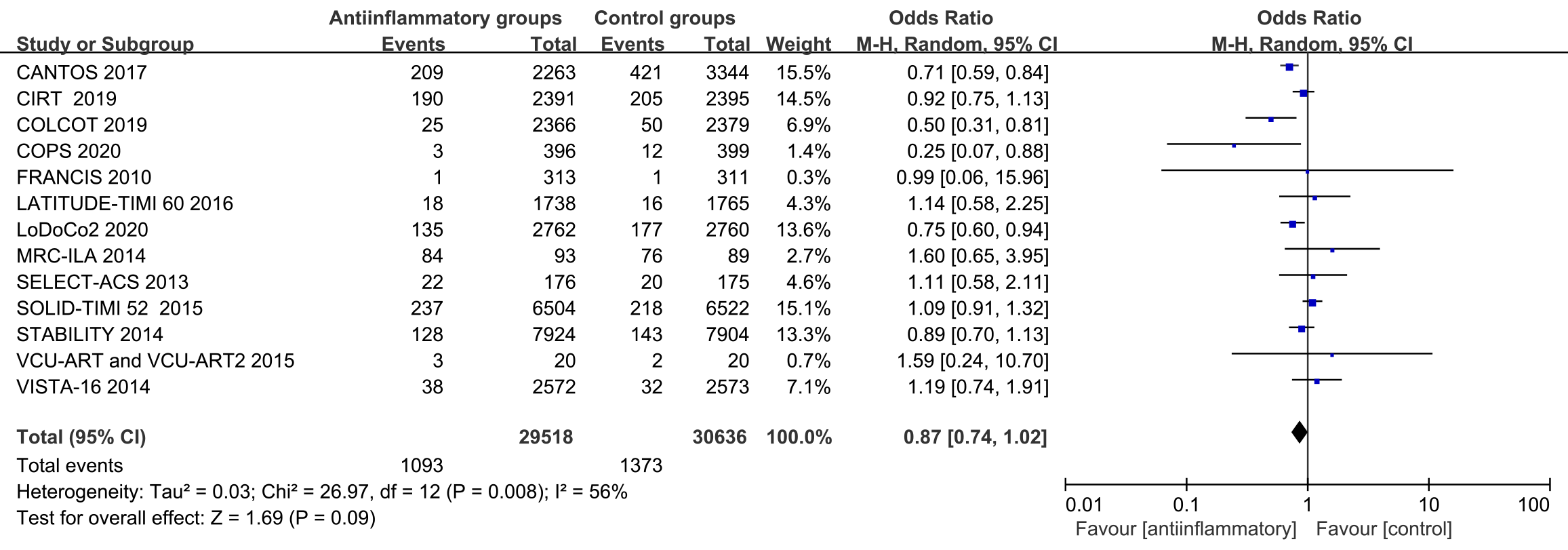

Supplement: Supplementary Figure 7 — Forest plots of studies evaluating revascularization in patients receiving anti-inflammatory agents vs. placebo. [file Image_7.TIF]

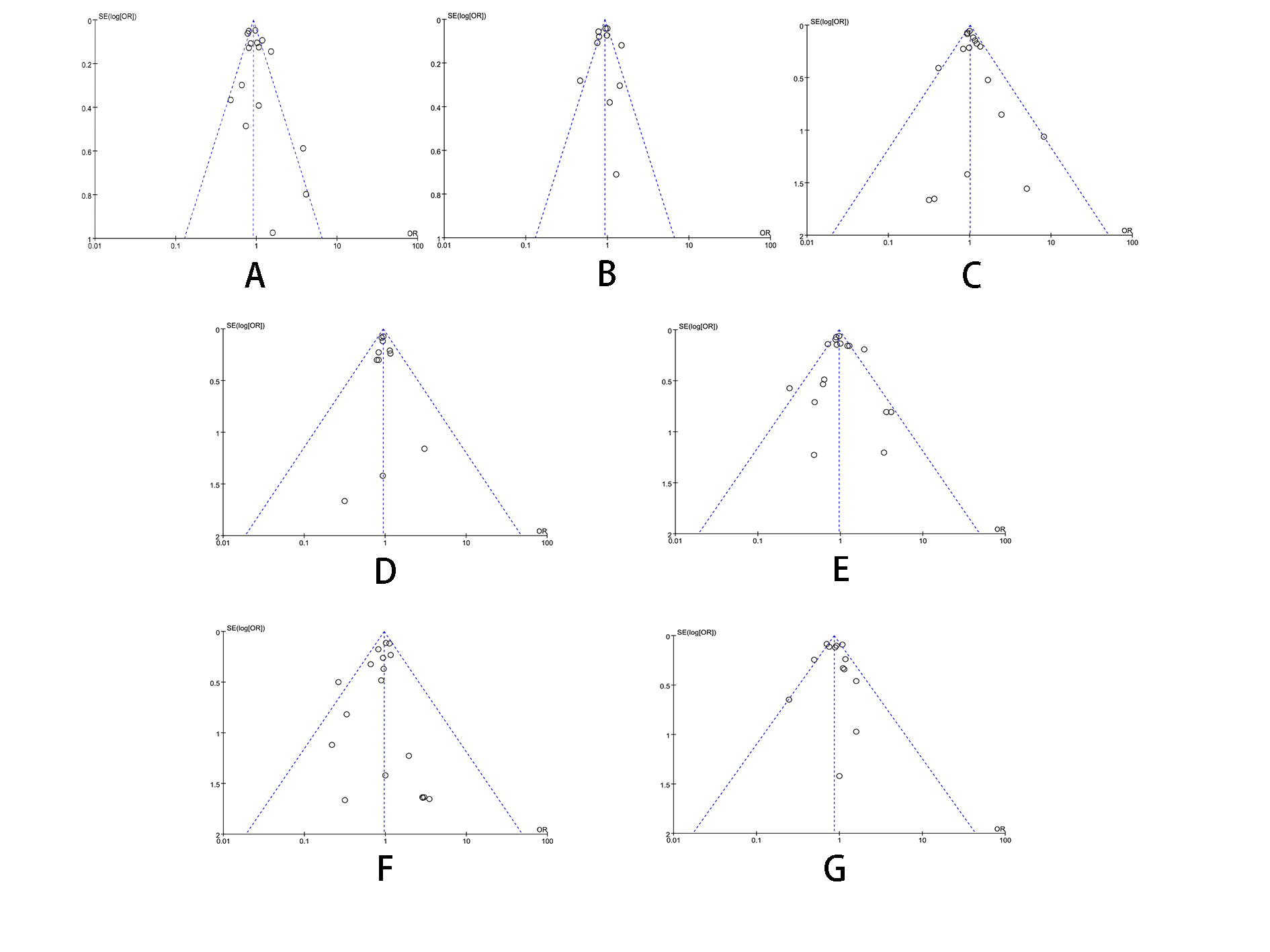

Supplement: Supplementary Figure 8 — Funnel plots of odds ratios and standard errors to assess the publication bias of anti-inflammatory agents vs. placebo in primary end points (A), secondary end points (B), all-cause mortality (C), cardiac mortality (D), recurrent myocardial infarction (E), stroke (F) and revascularization (G). [file Image_8.TIF]

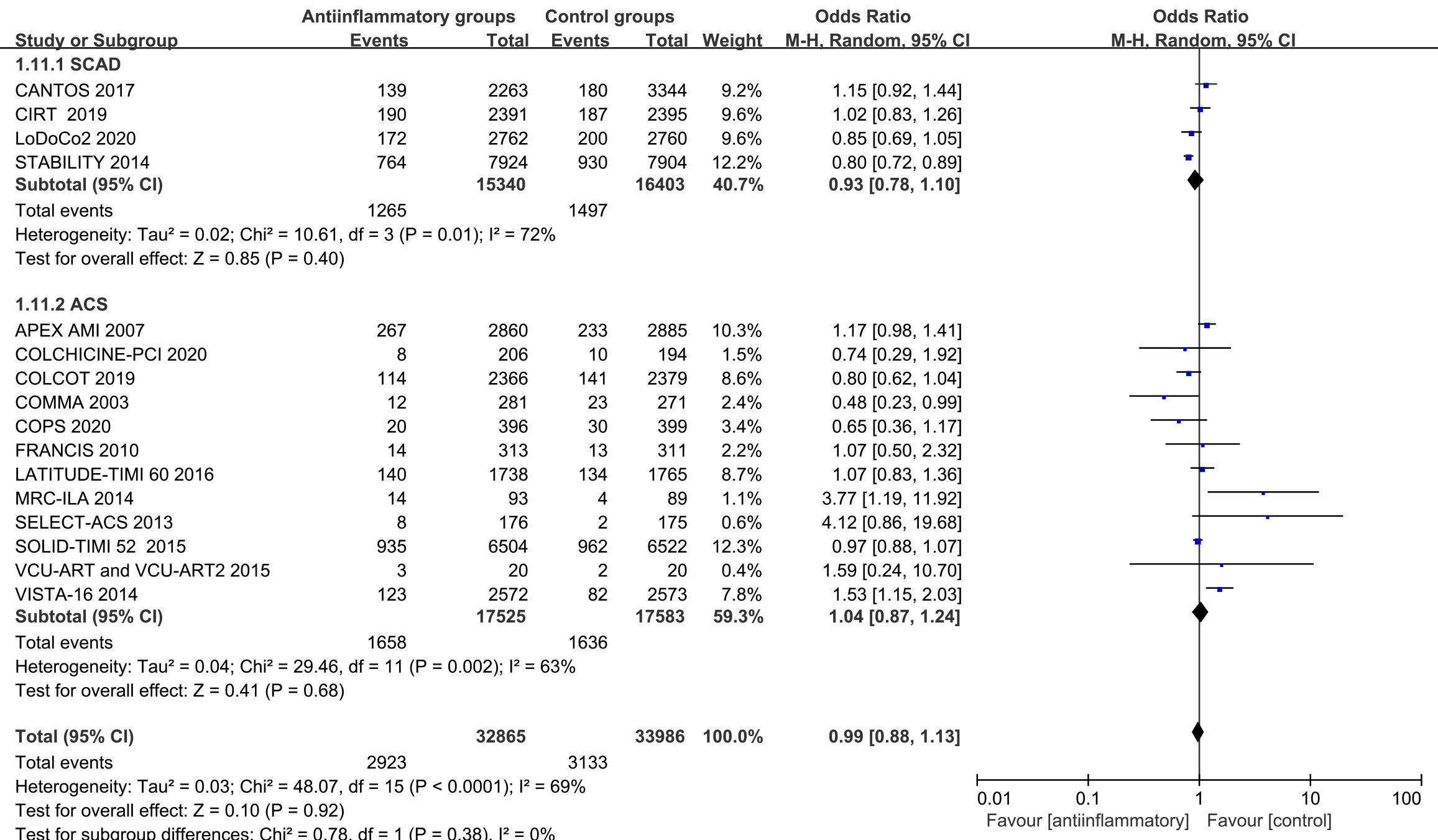

Supplement: Supplementary Figure 9 — Forest plots of studies evaluating primary end points in patients with stable CHD vs. ACS. [file Image_9.TIF]

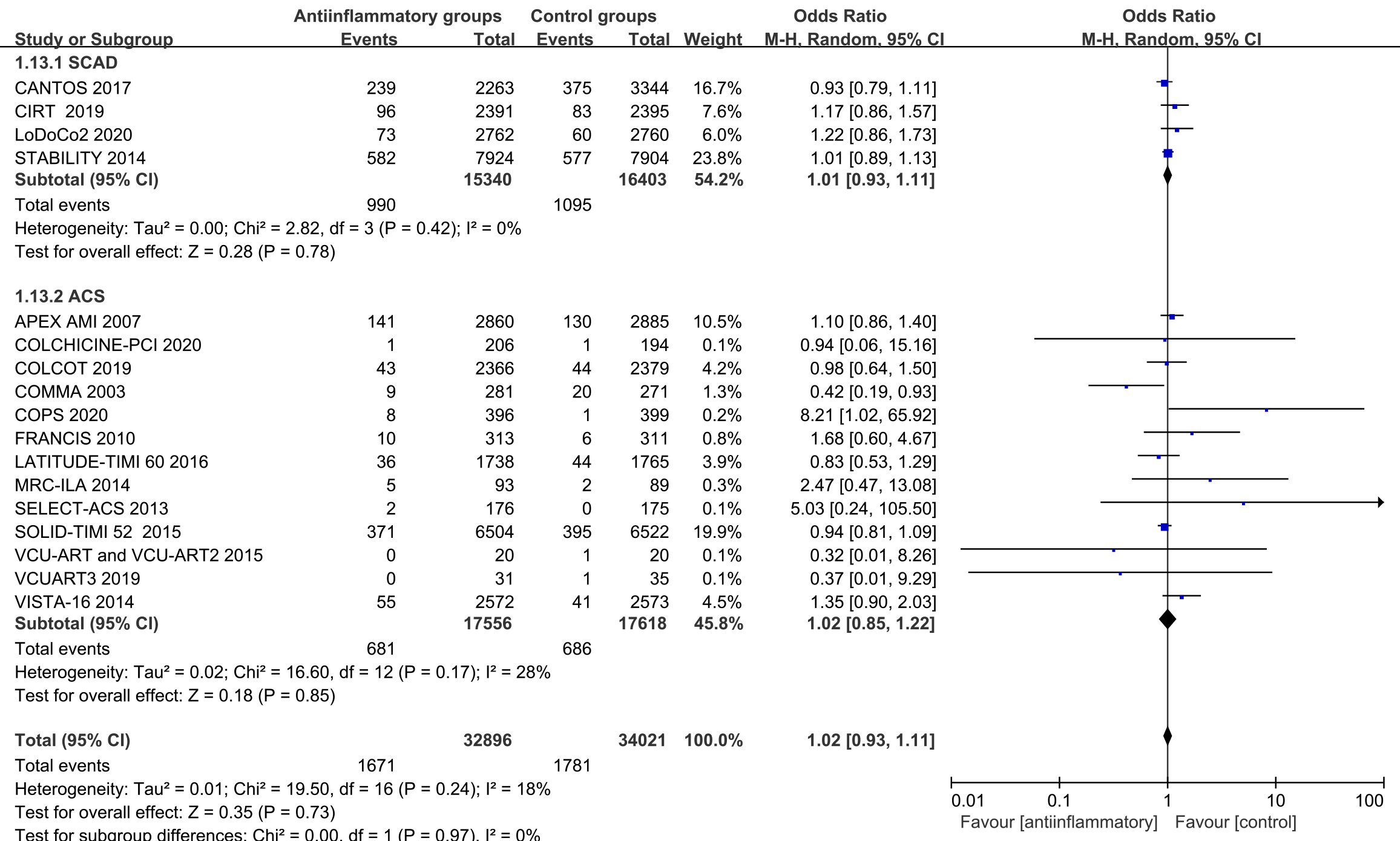

Supplement: Supplementary Figure 10 — Forest plots of studies evaluating all-cause mortality in patients with stable CHD vs. ACS. [file Image_10.TIF]

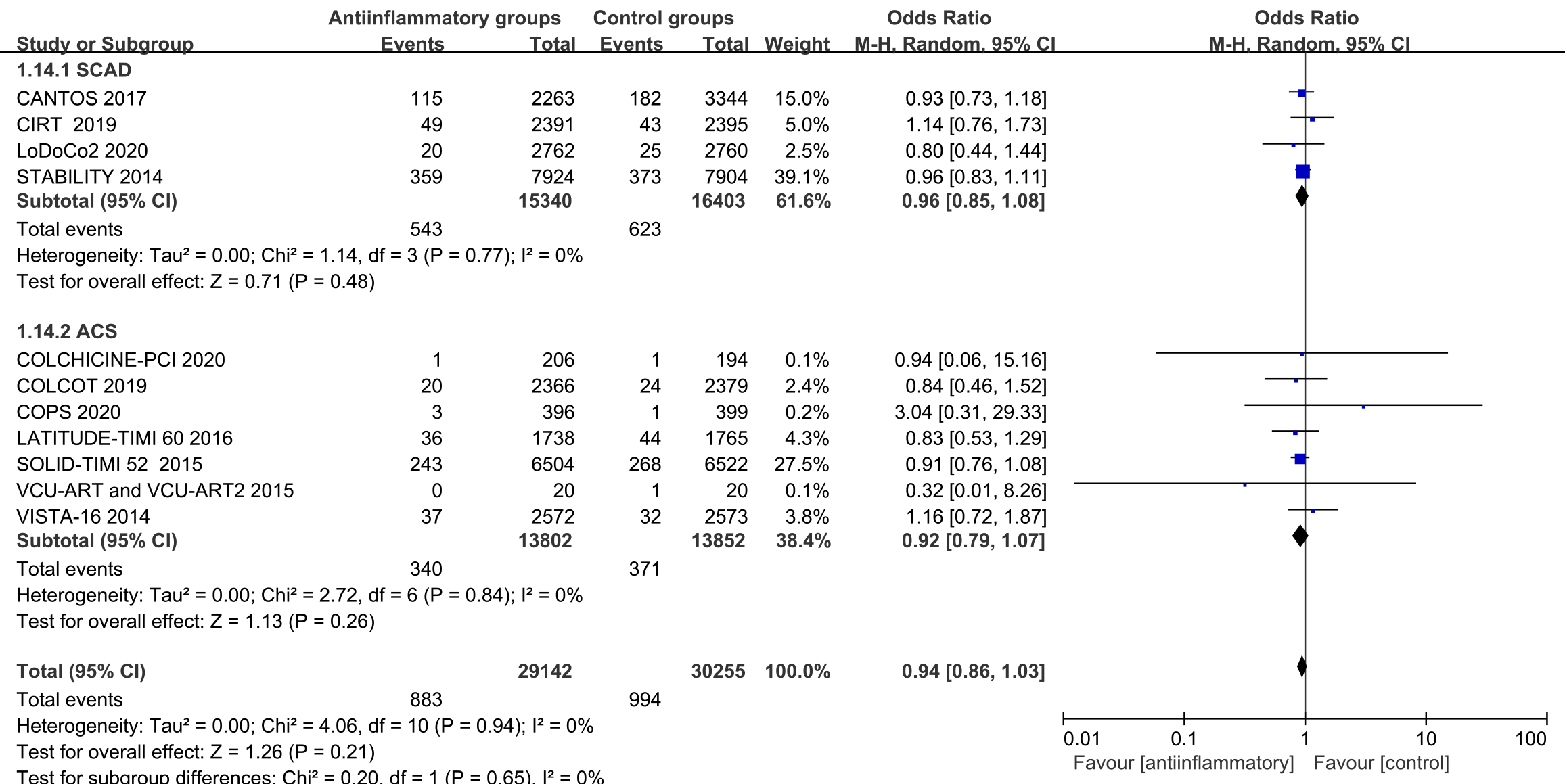

Supplement: Supplementary Figure 11 — Forest plots of studies evaluating cardiac mortality in patients with stable CHD vs. ACS. [file Image_11.TIF]

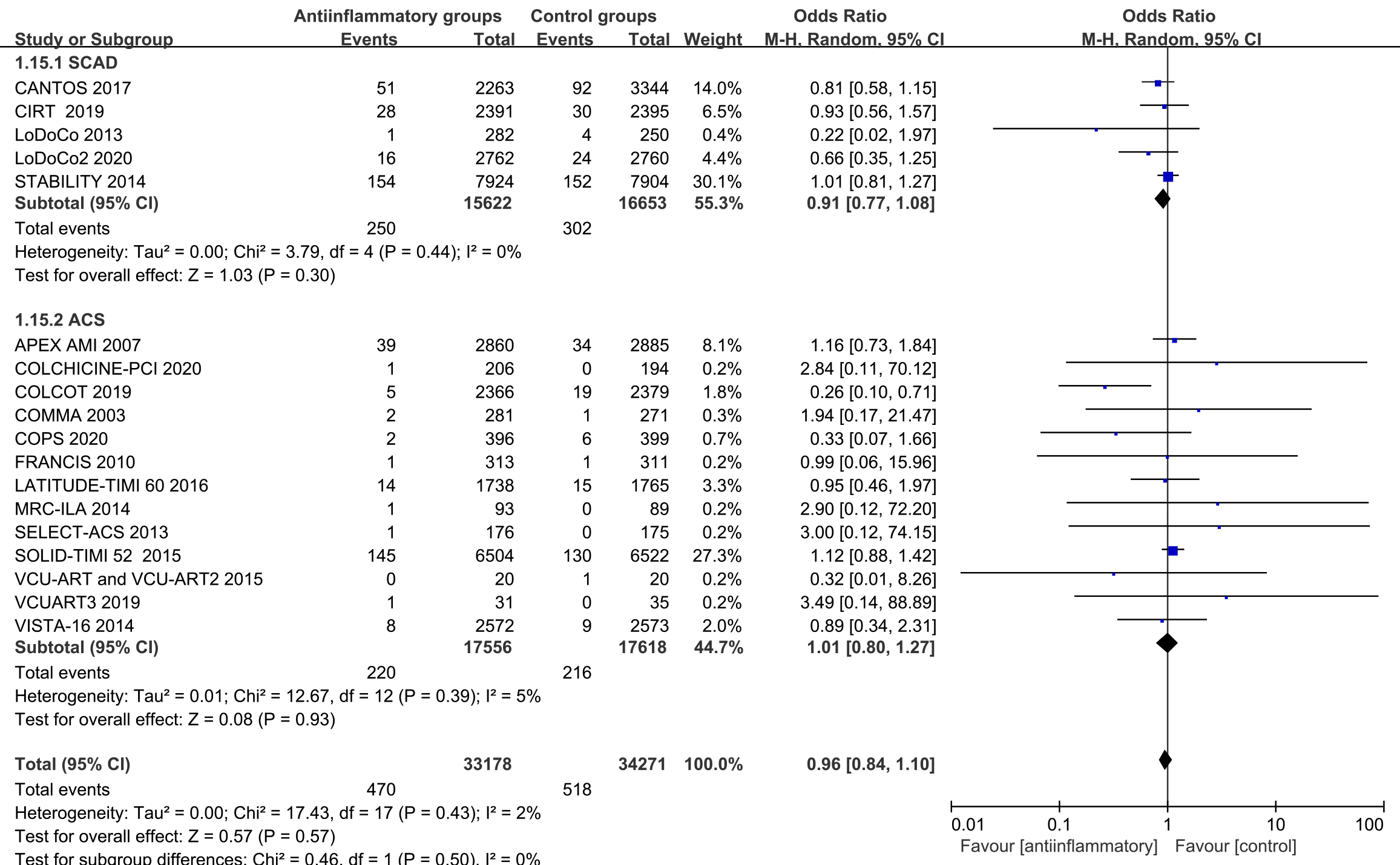

Supplement: Supplementary Figure 12 — Forest plots of studies evaluating stroke in patients with stable CHD vs. ACS. [file Image_12.TIF]

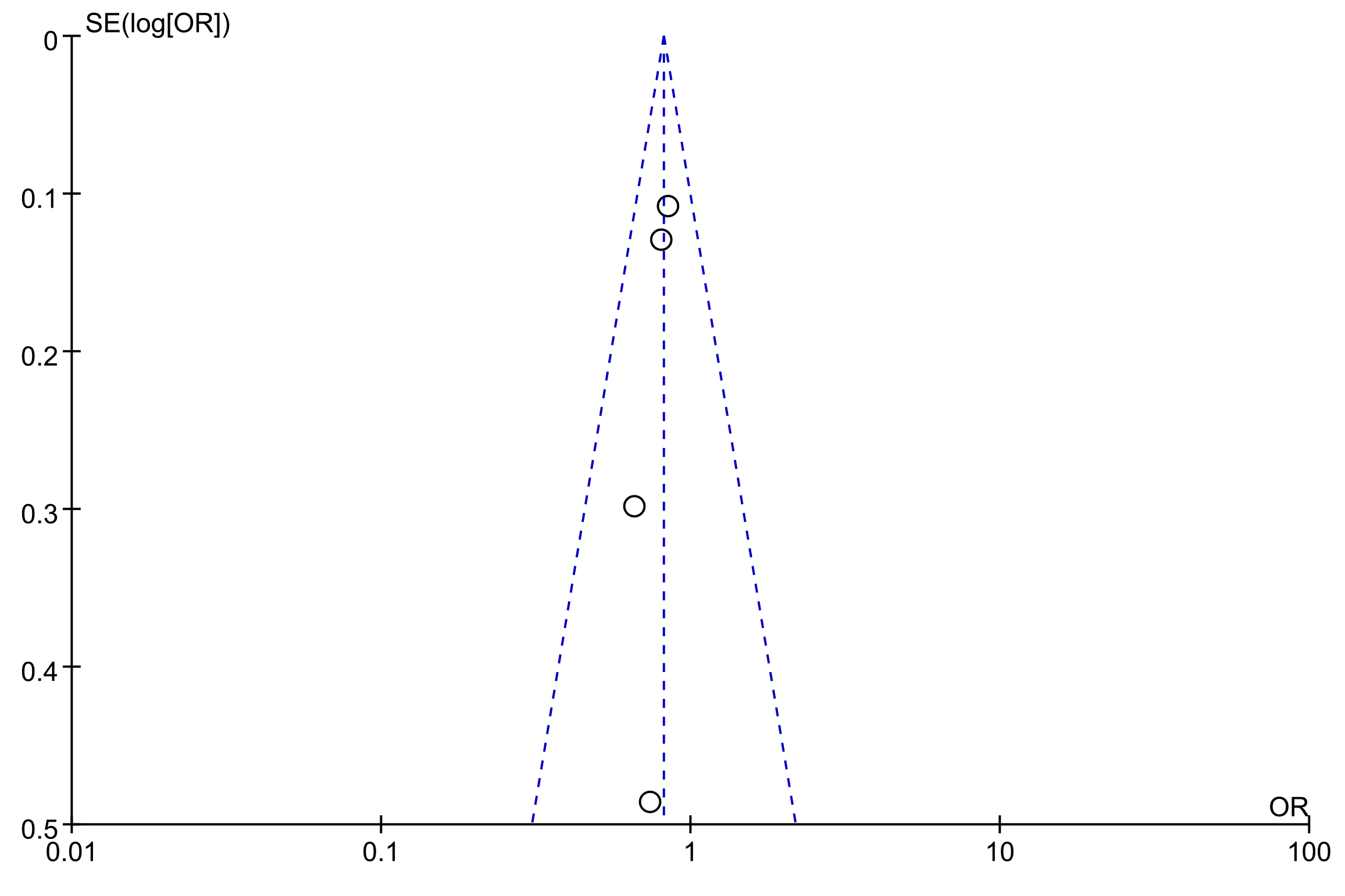

Supplement: Supplementary Figure 13 — Funnel plots of odds ratios and standard errors to assess the publication bias of colchicine vs. placebo in primary end points. [file Image_13.TIF]

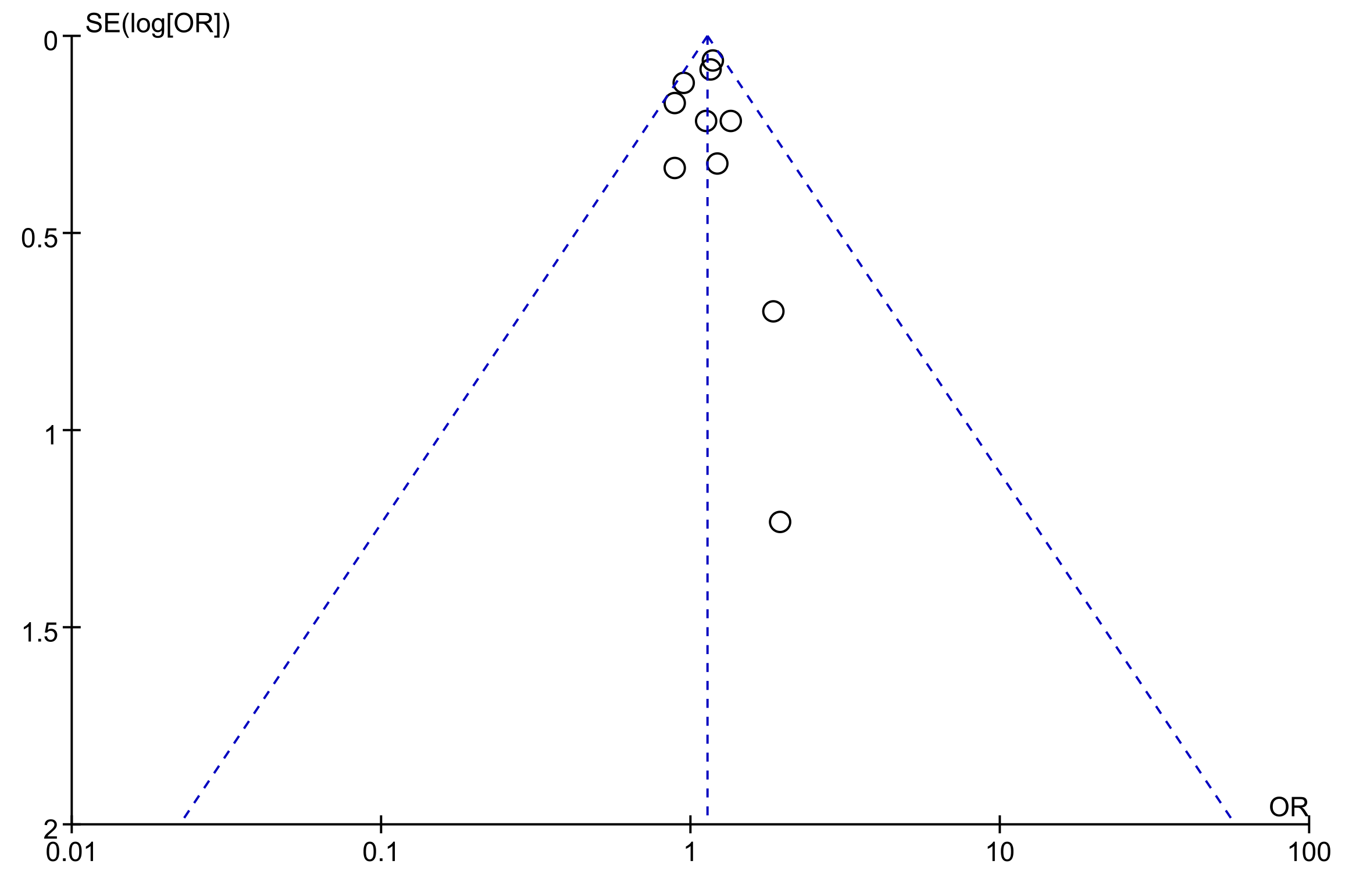

Supplement: Supplementary Figure 14 — Funnel plots of odds ratios and standard errors to assess the publication bias of anti-inflammatory agents vs. placebo in infections. [file Image_14.TIF]

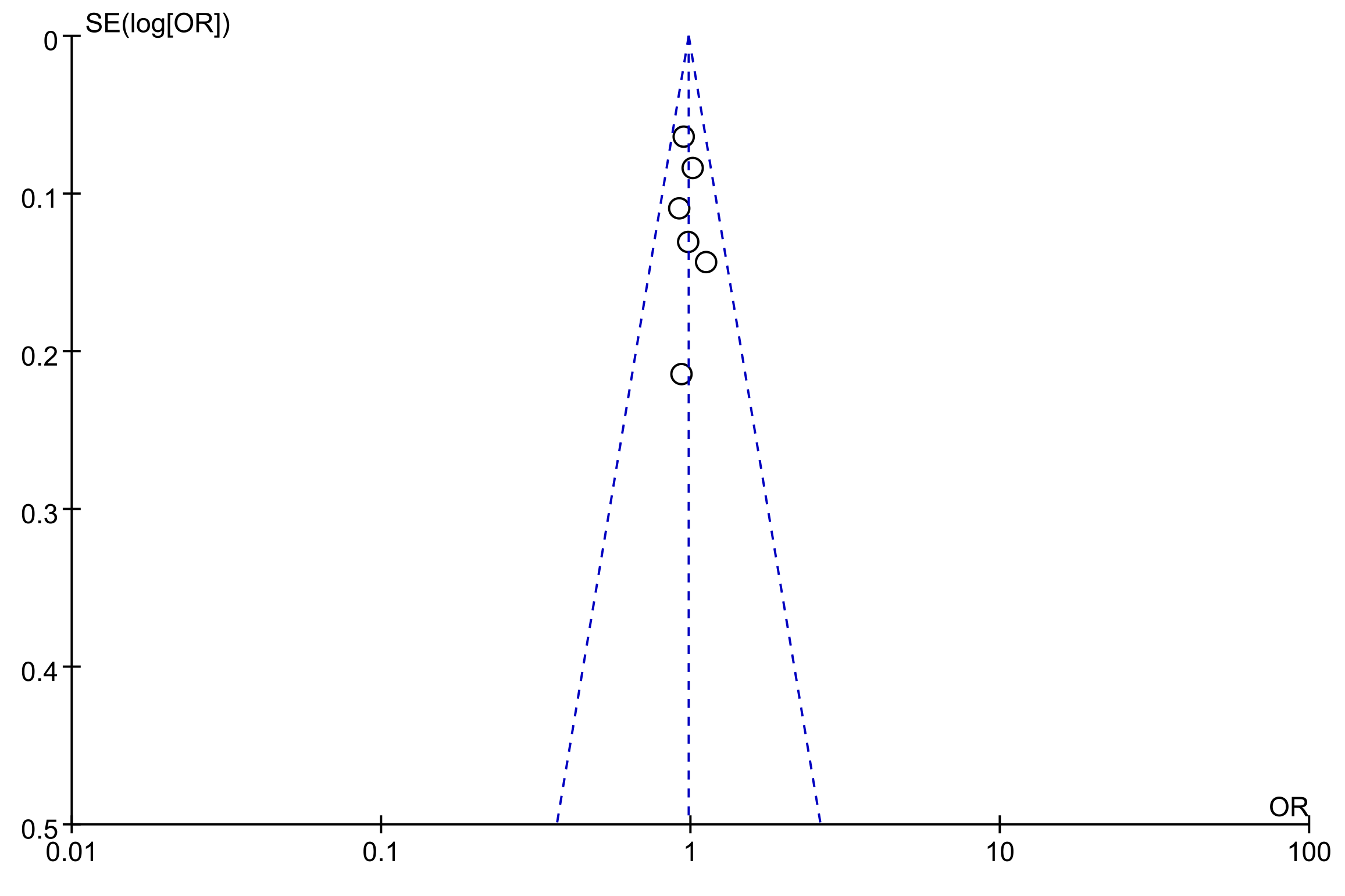

Supplement: Supplementary Figure 15 — Funnel plots of odds ratios and standard errors to assess the publication bias of anti-inflammatory agents vs. placebo in cancers. [file Image_15.TIF]
